# SID-2 is a conserved extracellular vesicle protein that is not associated with environmental RNAi in parasitic nematodes

**DOI:** 10.1098/rsob.240190

**Published:** 2024-11-06

**Authors:** Frances Blow, Kate Jeffrey, Franklin Wang-Ngai Chow, Inna A. Nikonorova, Maureen M. Barr, Atlanta G. Cook, Bram Prevo, Dhanya K. Cheerambathur, Amy H. Buck

**Affiliations:** ^1^ Institute of Immunology and Infection Research, School of Biological Sciences, University of Edinburgh, Edinburgh EH9 3JT, UK; ^2^ Wellcome Centre for Cell Biology & Institute of Cell Biology, School of Biological Sciences, University of Edinburgh, Edinburgh EH9 3BF, UK; ^3^ Department of Health Technology and Informatics, Hong Kong Polytechnic University, Hong Kong; ^4^ Department of Genetics and Human Genetics Institute of New Jersey, Rutgers University, Piscataway, New Jersey NJ 08854, USA; ^5^ Institute of Quantitative Biology, Biochemistry and Biotechnology, School of Biological Sciences, University of Edinburgh, Edinburgh EH9 3FF, UK

**Keywords:** extracellular vesicles, membrane protein, environmental RNAi, nematodes

## Abstract

In the free-living nematode *Caenorhabditis elegans,* the transmembrane protein SID-2 imports double-stranded RNA into intestinal cells to trigger systemic RNA interference (RNAi), allowing organisms to sense and respond to environmental cues such as the presence of pathogens. This process, known as environmental RNAi, has not been observed in the most closely related parasites that are also within clade V. Previous sequence-based searches failed to identify *sid-2* orthologues in available clade V parasite genomes. In this study, we identified *sid-2* orthologues in these parasites using genome synteny and protein structure-based comparison, following identification of a SID-2 orthologue in extracellular vesicles from the murine intestinal parasitic nematode *Heligmosomoides bakeri*. Expression of GFP-tagged *H. bakeri* SID-2 in *C. elegans* showed similar localization to the intestinal apical membrane as seen for GFP-tagged *C. elegans* SID-2, and further showed mobility in intestinal cells in vesicle-like structures. We tested the capacity of *H. bakeri* SID-2 to functionally complement environmental RNAi in a *C. elegans* SID-2 null mutant and show that *H. bakeri* SID-2 does not rescue the phenotype in this context. Our work identifies SID-2 as a highly abundant EV protein whose ancestral function may be unrelated to environmental RNAi, and rather highlights an association with extracellular vesicles in nematodes.

## 1. Introduction

Uptake of exogenous double-stranded RNA (dsRNA) and subsequent entry into RNA interference (RNAi) pathways enables organisms to sense and respond to environmental cues and is a potent mechanism of antiviral defence in some species [1]. Environmental RNAi has been characterized in the model nematode *Caenorhabditis elegans* using genetic screens [2,3] and the gene systemic RNA interference defective 2 (*sid-2*) was discovered as being essential for dsRNA import [4]. SID-2 is a single-pass transmembrane protein that is predominantly localized to the apical membrane of intestinal epithelial cells and is thought to play a role in the internalization of dsRNA through pH-dependent receptor-mediated endocytosis [5]. However, the precise mechanism by which SID-2 mediates dsRNA import and entry into RNAi pathways remains unknown. Although *sid-2* orthologues have been identified in many *Caenorhabditis* species, most are not capable of environmental RNAi, including one of the closest known phylogenetic relatives of *C. elegans*: *C. briggsae* [4,6].

Nematodes are an incredibly diverse and ubiquitous phylum that includes free-living species and species that parasitize plants, animals and humans [7]. *Heligmosomoides bakeri* is a murine gastrointestinal nematode that naturally infects house mice (*Mus musculus*) and belongs to the same phylogenetic clade as *C. elegans* (clade V) which also includes parasites infecting livestock and humans [8]. *H. bakeri* has been used as a laboratory model to study parasite manipulation of the host immune system due to its ability to cause chronic infections in mice via the secretion of immunomodulatory molecules [9]. Like most animal parasitic nematodes, *H. bakeri* is refractory to environmental RNAi [10]. This was previously hypothesized to be at least partially due to a lack of *sid-2* orthologues in this group of species [11,12]. We report here for the first time that *C. elegans sid-2* orthologues were identified in clade V parasitic nematodes using genome synteny and protein structure comparison tools in conjunction with chromosome-scaffolded genome assemblies. A phylogenetic analysis of *sid-2* orthologues from free-living and parasitic clade V nematodes revealed that the extracellular domain of SID-2 is highly divergent between species, in both free-living and parasitic nematodes.

In parallel, a recent study suggests SID-2 may play roles in other contexts, since it is released as an abundant cargo of ciliary extracellular vesicles (EVs) by *C. elegans* [13]. Previous studies have demonstrated a role of EVs released by *C.elegans* in worm-to-worm communication via manipulation of mating behaviours [13,14]. One way in which *H. bakeri* and other nematode parasites manipulate the host immune system is through the secretion of EVs bearing immunomodulatory cargo including small RNAs, which are taken up by host cells *in vitro* [15–18]. Based on a previous proteomic analysis, we found that, like *C. elegans* SID-2, *H. bakeri* SID-2 is abundant in EVs. This suggests that the conservation of SID-2 could relate to functions in EVs rather than environmental RNAi. We investigated the function of the SID-2 orthologue from the parasitic nematode *H. bakeri* by heterologous expression in *C. elegans*. We found that, as expected, *H. bakeri* SID-2 does not compensate for the *C. elegans* SID-2 environmental RNAi phenotype under endogenous *C. elegans* regulatory elements, indicating that the protein function at the intestinal luminal membrane is divergent between *C. elegans* and *H. bakeri* SID-2 orthologues. This work expands the known contexts in which SID-2 exists and introduces the question of whether an ancestral function of nematode SID-2 proteins could relate to cell–cell, nematode–nematode or nematode–host communication via EVs.

### 2. Methods

#### 2.1. *Sid-2* orthologue analysis

Genomic locations and gene accessions of *H. bakeri* and *C. elegans sid-2* orthologues are listed in table 1, and the corresponding amino acid sequences are available in electronic supplementary material, table S2. Signal peptides and transmembrane domains were predicted using DeepTMHMM with default settings [19]. AlphaFold structures were predicted using AlphaFold 3 with default settings [20]. N-glycosylation sites were predicted using NetNGlyC version 1.0 with default settings [21].

Orthologues of *H. bakeri sid-2* HPOL_0001199201 were identified in 14 species of clade V parasitic nematodes using the ‘Orthologues’ function in WormBase ParaSite [22] and visually inspected to be nested within a *dyf-2* orthologue. Truncated sequences with length less than 40% of HPOL_0001199201 (358 aa) were excluded from further analyses. We used the protein-to-genome alignment tool miniprot version 0.13 [23] to identify orthologues of HPOL_0001199201 in the genomes of three further species of clade V parasitic nematode that are not currently available in WormBase ParaSite: *Teladorsagia circumcincta*, *Trichostrongylus colubriformis* and *Heligmosomoides polygyrus* (genome accessions are listed in electronic supplementary material, table S2). *Caenorhabditis* nematode orthologues of *C. elegans sid-2* (WBGene00004796) were identified using the ‘Orthologues’ function in WormBase [24]. Details and amino acid sequences of *sid-2* orthologues from clade V parasitic and *Caenorhabditis* nematodes are listed in electronic supplementary material, table S2.

The conservation score among clade V nematodes for each amino acid residue in *H. bakeri* SID-2 (HPOL_0001199201) was calculated using the Bayesian method implemented in the ConSurf server [25]. A custom multiple sequence alignment (electronic supplementary material, file S1) was generated using Clustal Omega with default settings [26] and manually inspected. ConSurf analysis was performed twice to identify regions of HPOL_0001199201 that are conserved with *Caenorhabditis* and other parasitic clade V nematodes: firstly, with all 28 single-copy *sid-2* sequences detailed in electronic supplementary material, table S2; secondly, with only the 13 *sid-2* sequences from parasites detailed in electronic supplementary material, table S2. ConSurf conservation scores were plotted in R Studio [27] using R version 4.4.0 [28] with the packages ggplot2 [29], ggforce [30] and tidyr [31]. The neighbour-joining tree generated by ConSurf was visualized in R using the packages ggtree version 3.12.0 [32] and treeio version 1.28.0 [33] (electronic supplementary material, figure S2A), and the *H. bakeri* AlphaFold model was visualized with ConSurf conservation scores using PyMol (Schrödinger LLC) (electronic supplementary material, figure S2B).

#### 2.2. *H. bakeri* extracellular vesicle purification

*H. bakeri* EVs were purified from adult *H. bakeri* excretory–secretory products (HES) collected up to 8 days post-*in vitro* culture as described previously [17]. Eggs and debris were removed from HES by spinning at 400*g* for 5 mins at RT and then filtered using a 0.22 μm filter. Filtered HES was concentrated using a VivaSpin 20 centrifugal concentrator with a 5 kDa molecular weight cutoff (Sartorius). EVs were purified from concentrated HES by ultracentrifugation at 100 000*g* for 90 mins at 4°C in polyallomer tubes (Beckman Coulter) in a SW40 rotor (Beckman Coulter). The supernatant (EV-depleted HES) was removed and concentrated using a VivaSpin 20 centrifugal concentrator with a 5 kDa molecular weight cutoff (Sartorius). Purified EVs were washed twice with PBS (Sigma-Aldrich) and pelleted each time by ultracentrifugation at 100 000*g* for 70 mins at 4°C in polyallomer tubes (Beckman Coulter) in a SW40 rotor (Beckman Coulter). The pellet was resuspended in PBS and EV particle size and counts were measured using a Zetaview TWIN particle tracking analyser (Particle Metrix), and protein concentrations were measured using the Qubit Protein Assay (Qubit). EVs were aliquoted and stored at −80°C until use.

#### 2.3. Silver stain

Samples containing 1 μg total protein were separated by SDS-PAGE and incubated with fixing solution (40% ethanol, 10% glacial acetic acid) for 2 h 30 mins. Gels were incubated with sensitization solution (30% ethanol, 0.2% sodium thiosulfate, 6.8% sodium acetate) for 30 mins, followed by 0.25% silver nitrate solution for 20 mins. Gels were developed with developing solution (2.5% sodium carbonate, 20 μl 37% formaldehyde) for 2 mins, incubated with stopping solution (50 mM EDTA) for 10 mins, and imaged using a ChemiDoc gel imager (BioRad).

#### 2.4. Western blot

Samples containing 1 μg total protein were separated by SDS-PAGE, transferred to a PVDF membrane and incubated overnight at 4°C with polyclonal antibodies raised in rabbits against the *H. bakeri* SID-2 extracellular domain peptide CSNRVPSGQDDKNITVT (Sino Biological). Goat anti-Rabbit IgG (H+L) secondary antibody (Invitrogen, SA5-35571) was used, and membranes were visualized on a LiCor Odyssey imager (LiCor).

#### 2.5. *C. elegans* strains

The *C. elegans* strains were grown on nematode growth medium (NGM) plates seeded with *E. coli* OP50 bacteria at 20°C. All strains used in the study are listed in electronic supplementary material, table S3.

#### 2.6. *C. elegans* transgenic strain construction

The Mos1 mediated single copy insertion MosSCI method [34] was used to generate transgenic animals stably expressing (CeSID-2::GFP and HbSID-2::GFP) transgenes under the control of the *cesid-2* promoter (2 kb upstream of the ATG start codon of C. elegans *sid-2* gene locus) and *unc-54* 3′ UTR. This method, described by Frøkjaer-Jensen *et al*. [34], is a well-established transposon-based strategy utilized for the targeted integration of desired transgenes into specific, well-characterized and innocuous genomic loci. Two plasmids, pDC1223 (Cel-SID-2pro::CeSID-2::GFP::unc-543'UTR) and pDC1210 (Ce-SID-2pro::HbSID-2::GFP::unc-54 3'UTR) expressing CeSID-2::GFP and HbSID-2::GFP, respectively, were constructed with their regulatory sequences and cloned into the pCFJ151 vector using Gibson assembly [35]. These constructs were then inserted into the chromosome II (*ttTi5605*) locus harbouring the *Drosophila* Mos1 element, which is cleaved by Mos1 transposase. The pCFJ151 vector comprises homology arms that direct transgene integration into the ttTi5605 mos locus and a positive selection marker cassette for the *Caenorhabditis briggsae* unc-119 gene (Cb-unc-119). A mixture of plasmids, including the repair plasmid with the transgene of interest and Cb-unc-119 positive selection marker (50 ng µl^−1^), the transposase plasmid (pCFJ601, Peft-3::Mos1transposase, 50 ng µl^−1^) and four negative selection marker plasmids (pCFJ90 (Pmyo-2::mCherry, 2.5 ng µl^−1^), pCFJ104 (Pmyo-3::mCherry, 5 ng µl^−1^), pGH8 (Prab-3::mCherry, 10 ng µl^−1^) and pMA122 (Phsp-16.41::peel-1, 10 ng µl^−1^)), were injected into the gonad of young adult unc-119 mutant animals harbouring the ttTi5605 mos locus. The pCFJ90, pCFJ104 and pGH8 plasmids are fluorescent markers that select against extrachromosomal arrays, while pMA122 encodes the toxic protein PEEL-1 under a heat shock promoter. Following injection, the animals were allowed to produce F1 and F2 progeny. After one week, the progeny of the injected worms were heat-shocked at 34°C for 2–4 h to induce PEEL-1 expression, eliminating worms carrying extrachromosomal arrays. Moving, non-fluorescent worms were then selected, and insertions were confirmed by PCR using primers spanning both homology arms. Both CeSID-2::GFP and HbSID-2::GFP expressing transgenic strains were then crossed into the sid-2 null strain background (PT3646) using standard genetic methods.

#### 2.7. RNAi assay

To perform RNAi-mediated depletion, we designed the targeting sequence for *hcp-4* to be at nucleotide positions 967–2128 after the first ATG codon, as described by Taylor *et al*. [36]. The *hcp-4* targeting sequence was inserted into the L4440 plasmid and transformed into HT115 (*DE3*) bacteria [37]. Bacterial clones containing the RNAi sequence were cultured overnight at 37°C in LB medium with 100 μg ml^−1^ ampicillin. Saturated cultures were diluted 1:100 and grown until reaching an OD600 of 0.8–1. Isopropyl-β-D-thiogalactopyranoside (IPTG) was added to a final concentration of 1 mM, and the cultures were incubated for 1 h at 37°C. The bacteria were then seeded onto NGM plates containing agarose and 1 mM IPTG, and the plates were allowed to dry. L4 worms were subsequently plated on RNAi plates, maintained at 20°C and the RNAi assay was performed as outlined in figure 4*a* and as follows.

On experimental day 1, a single L4 hermaphrodite of the desired strain was placed onto each seeded plate (*n* = 10). After 24 h (day 2), the same animal (now an adult) was transferred to a fresh seeded plate. This process was repeated every 24 h, moving the animal to a new plate on day 3 and removing it on day 4. Following the removal of the animal, each plate was incubated at 20°C for 48 h before counting the number of live L4 progeny and dead/unhatched eggs. The number of dead eggs was added to the number of live offspring for each plate, and the percentage viability of the strain after *hcp-4* dsRNA ingestion was determined by calculating the number of live progeny divided by the total progeny. Due to heterogeneity of variance between strains and different sample sizes (Ns) in each cohort, percentage viability of offspring in the RNAi assay was analysed using a Kruskal–Wallis test followed by Dunn’s test for post hoc comparisons between strains. Statistical analyses were performed in GraphPad prism version 10.2.3 (GraphPad Software) with a significance *α* threshold of 0.05.

#### 2.8. *C. elegans* live imaging

For all imaging experiments, L4 animals were anaesthetized using 5 mM levamisole and mounted in M9 on 2% agarose pads. Images were acquired with a CFI60 Plan Apochromat lambda 100× (Nikon) objective mounted on a spinning disc confocal microscopy system. The system was equipped with a Yokogawa spinning disk unit (CSU-W1), a Nikon Ti2-E fully motorized inverted microscope, and a Photometrics Prime 95B camera.

To image the localization of CeSID-2::GFP and HbSID-2::GFP in the gut, a single *z*-slice of the gut was focused at the centre for still images. For time-lapse movies, a single z-slice of the apical surface of the gut was captured every 2 s for a total duration of 2 min. All acquired images were then processed using ImageJ (Fiji) software.

### 3. Results

#### 3.1. Identification of an *H. bakeri* protein orthologous to *C. elegans* SID-2 that is enriched in extracellular vesicles

**Figure 1.**
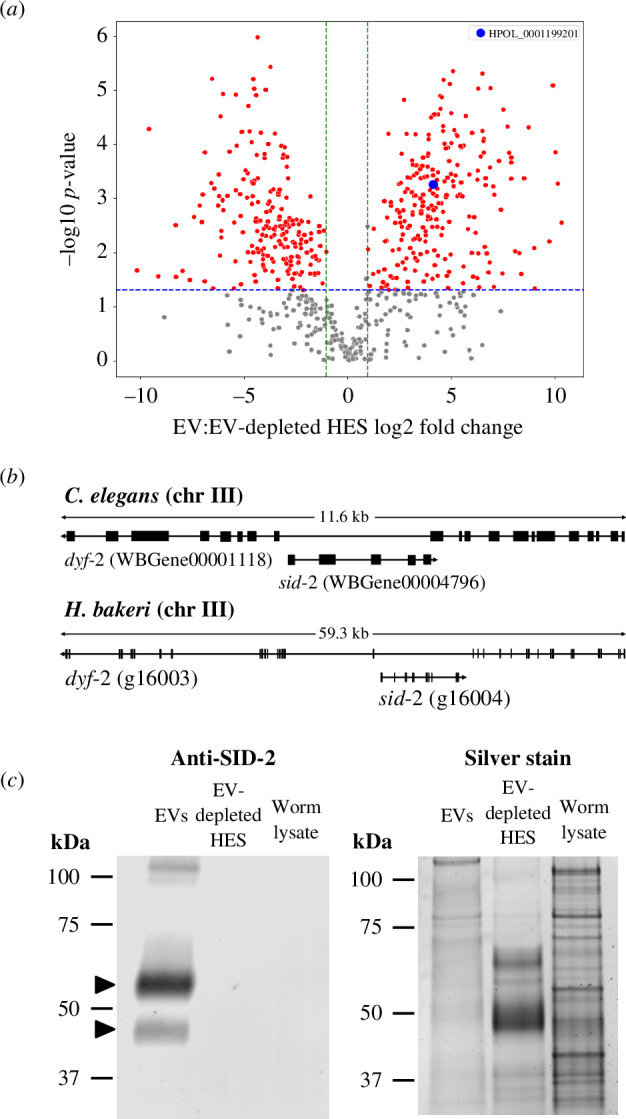
The parasitic nematode *H. bakeri* encodes an orthologue of *C. elegans sid-2* that is enriched in extracellular vesicles released by the parasite. (*a*) Scatter plot of proteins enriched in EVs compared with EV-depleted HES identified by LC-MS/MS (from [15]). The *sid-2* orthologue, HPOL_0001199201, is highlighted in blue. Red points indicate *p *< 0.05 and log2 fold change (FC)>1, while grey points indicate non-significant proteins. The blue horizontal dashed line represents the *p*-value threshold of 0.05, and the green vertical dashed lines represent log2 FC thresholds of 1 and −1. (*b*) Genome synteny of *sid-2* orthologues on chromosome III in *C. elegans* and *H. bakeri*. In both genomes, *sid-2* is encoded on the opposite strand in an intron of a larger gene *dyf-2*. Black boxes indicate exons, and genes are displayed from start codon to stop codon. Arrows indicate gene orientation in the genome. (*c*) Equal amount of total protein (1 μg) isolated from *H. bakeri* adult worm lysate, EVs and EV-depleted HES was assayed by anti-SID-2 western blot (left panel) and silver stain (right panel). SID-2 bands approximately 45 kDa and approximately 55 kDa enriched in EVs are marked with black triangles on the anti-SID-2 western blot (left panel).

Our previous proteomic analysis identified proteins enriched in EVs compared with EV-depleted *Heligmosomoides* excretory–secretory products (HES) from *in vitro* cultured *H. bakeri* adult worms [15]. Several of these EV-enriched proteins have no annotated function (electronic supplementary material, table S1). One of the abundant EV proteins, HPOL_0001199201 (WormBase ParaSite accession), which exhibits a 17.5-fold enrichment in EV fractions compared with non-EV fractions in the proteomic analysis (figure 1*a*; electronic supplementary material, table S1), was analysed using BLASTp [38]. The search was conducted against *C. elegans* annotations in WormBase and identified SID-2 (protein accession ZK520.2) as a potential orthologue by a low-identity sequence match.

Because genome synteny is highly conserved in clade V nematodes [39], we compared the genomic locations of *C. elegans sid-2* and *H. bakeri* HPOL_0001199201. In *C. elegans* genome assembly WBcel235 on WormBase (version WS290) *sid-2* (WBGene00004796) is nested within the larger gene *dyf-2* (WBGene00001118) on chromosome III (table 1, figure 1*b*). Similarly, HPOL_0001199201 (annotated as g16004 in an updated chromosome-scale genome assembly for *H. bakeri* nxHelBake1.1 [40]) is nested within a larger gene *dyf-2*-like (g16003) on chromosome III (table 1, figure 1*b*). Amino acid identity between *C. elegans* SID-2 and HPOL_0001199201 is only 24.5% (electronic supplementary material, figure S1A). However, similarities in the AlphaFold models generated using AlphaFold 3 [20] for HPOL_0001199201 (electronic supplementary material, figure S1B) and *C. elegans* SID-2 (electronic supplementary material, figure S1C) indicate structural similarity such as the conserved barrel in the extracellular domain of both proteins, the single-pass transmembrane alpha helix, and the disordered cytoplasmic domain, although low confidence for some regions of the models. Similarly, a FoldSeek [41] search of the *C. elegans* SID-2 AlphaFold model against the AlphaFold database restricted to *H. bakeri* proteins returns HPOL_0001199201 as the top hit, with an e-value of 1.2 × 10^−8^. Both models have a similar arrangement of a folded beta-barrel domain followed by a likely transmembrane helix of approximately 40 residues. Superposition of the beta barrel domains of the highest ranking models using GESAMT [42] implemented in the CCP4i suite [43] gives a root mean square deviation (r.m.s.d.) of 2.11 Å over 75 C-alpha atoms and a quality score of 0.44 (where scores >0.1 indicate good structural similarity, a score of 1.0 would indicate an identical structure). Given the shared genome synteny and protein structural similarity of HPOL_0001199201 and *C. elegans sid-2*, we hereafter refer to HPOL_0001199201 as *H. bakeri sid-2*.

**Table 1.** Details of genomic loci encoding *H. bakeri* and *C. elegans sid-2* orthologues.

organism	genome assembly name	gene accession	chromosome	gene start coordinates	gene end coordinates	citation
*H. bakeri*	nxHelBake1.1	g16004	III	1 38 99 061	1 39 07 575	[40]
*C. elegans*	WBcel235 version WS290	WBGene00004796	III	1 36 79 445	1 36 82 450	[24]

Consistent with the proteomic analysis, western blot analysis using an antibody raised against *H. bakeri* SID-2 confirms enrichment of SID-2 in EVs when loading equal amounts (1 μg) of total protein from *H. bakeri* adult worm lysate, EVs (approx. 2.4 × 10^9^ particles loaded) and EV-depleted HES (figure 1*c*; left panel). *H. bakeri* SID-2 has a predicted molecular weight of 39 kDa but migrates at approximately 45 kDa and approximately 55 kDa (marked with black triangles) in the EV sample, with neither band observed in EV-depleted HES or worm lysate. The disparity in predicted and observed molecular weight may relate to modifications such as glycosylation (*H.bakeri* SID-2 is predicted to have three N-glycosylation sites by NetNGlyc version 1.0) and/or altered migration as a transmembrane protein [44,45]. Silver stain (figure 1*c*, right panel) further confirms that worm lysate, EV and EV-depleted HES had sufficient protein loaded.

#### 3.2. Clade V nematode parasites encode orthologues of *C. elegans sid-2*

**Figure 2.**
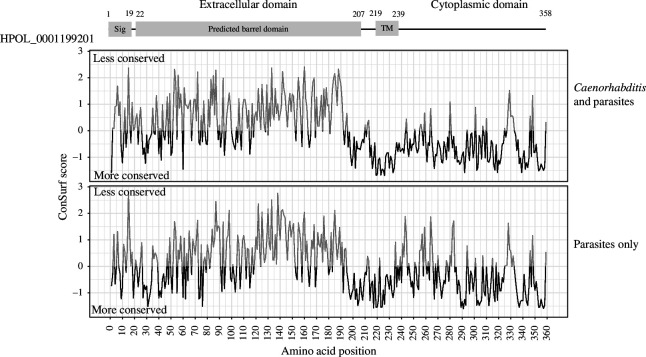
ConSurf analysis of amino acid conservation of HPOL_0001199201 with *sid-2* orthologues from 28 species of clade V nematode including both *Caenorhabditis* and parasitic species (top), and just parasitic species (bottom). Negative values (black) indicate residue conservation and positive values (grey) indicate residue variability at that position. The predicted structure of the protein is displayed at the top of the figure.

Previous homology-based searches failed to detect orthologues of *C. elegans sid-2* in clade V parasitic nematodes [12]. Using the conserved synteny of *sid-2* nested within *dyf-2* (figure 1*b*) and recently improved genomes for clade V parasitic nematodes, we identified orthologues of *H. bakeri sid-2* in clade V parasitic nematodes using the ‘Orthologues’ feature in WormBase ParaSite and the gene finding tool miniprot version 0.13 [23] (electronic supplementary material, table S2). Orthologues of *C. elegans sid-2* were identified in *Caenorhabditis* nematodes using the ‘Orthologues’ function in WormBase (electronic supplementary material, table S2). ConSurf analysis [25,46,47] of all 28 amino acid sequences from both groups was performed on the multiple sequence alignment (electronic supplementary material, file S1) to identify regions of the protein that are conserved in clade V nematodes. This indicates that the cytoplasmic and transmembrane domains of *sid*-2 orthologues are more highly conserved than the extracellular domain, and that this is not unique to parasites, but is seen across all clade V nematode *sid-2* orthologues studied (figure 2; electronic supplementary material, figure S2B).

#### 3.3. *H. bakeri* SID-2 localizes to the intestinal apical membrane and mobile vesicle-like structures when expressed in *C.elegans*

**Figure 3.**
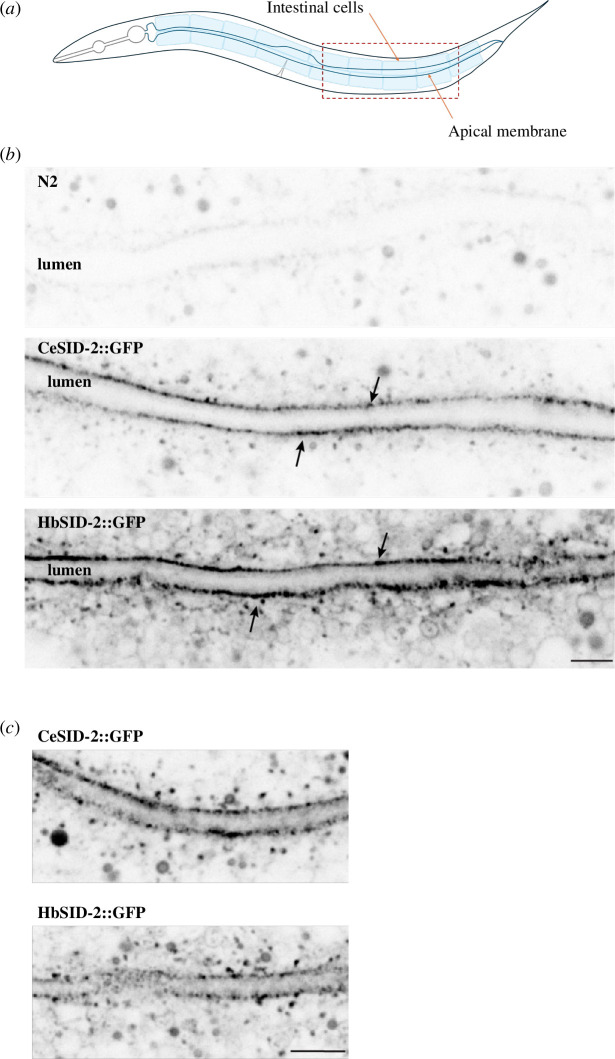
Expression of SID−2::GFP transgenes. (*a*) Schematic of the intestine in *C. elegans*. (*b*) Images of *C. elegans* intestine in N2 (control) animals and animals expressing CeSID-2::GFP and HbSID-2::GFP transgenes. The black arrows point to the apical surface of the intestine. Scale bar, 5 μm. (*c*) Apical surface view of the *C. elegans* intestine. Orange arrows point to vesicle-like structures budding off from the apical surface in animals expressing CeSID-2::GFP and HbSID-2::GFP transgenes. Scale bar, 2.5 μm.

To test the ability of *H. bakeri* SID-2 to compensate the function of *C. elegans* SID-2 in environmental RNAi, we generated a transgenic *C. elegans* SID-2 null strain expressing a single copy of N-terminally GFP-tagged *H. bakeri* SID-2 under control of the *C. elegans sid-2* promoter (HbSID-2::GFP, strain DKC1285). As a positive control, we generated a SID-2 null strain expressing a single copy of N-terminally GFP-tagged *C. elegans* SID-2 under control of the *C. elegans sid-2* promoter using the same method (CeSID-2::GFP, strain DKC1365). Imaging of the intestine of the transgenic strains shows similar subcellular localization between the *H. bakeri* and *C. elegans* SID-2 proteins, localizing to the apical membrane of all intestinal cells (figure 3*a,b*) and when zoomed-in GFP-fluorescence appears as cytoplasmic vesicles (figure 3*c*). Imaging of the wild-type N2 strain showed some autofluorescence in the intestine, as expected, but not at the apical luminal membrane (figure 3*b*). The expression in both strains is comparable with the apical membrane localization seen in *C. elegans* with fluorescent tags fused to SID-2 in the endogenous locus via CRISPR-Cas9 modification [13], indicating that transgenic proteins are localized similarly to endogenous *C. elegans* SID-2.

We also observed GFP fluorescence and movement consistent with vesicles trafficking in the intestine. This was evident for transgenic strains expressing either *C. elegans* (electronic supplementary material, movie S1) or *H. bakeri* (electronic supplementary material, movie S2) SID-2, suggesting that properties of SID-2 dictating its localization and mobility are conserved in *C. elegans* and *H. bakeri*.

#### 3.4. *H. bakeri* SID-2 does not compensate for *C. elegans* SID-2 function in environmental RNAi

**Figure 4.**
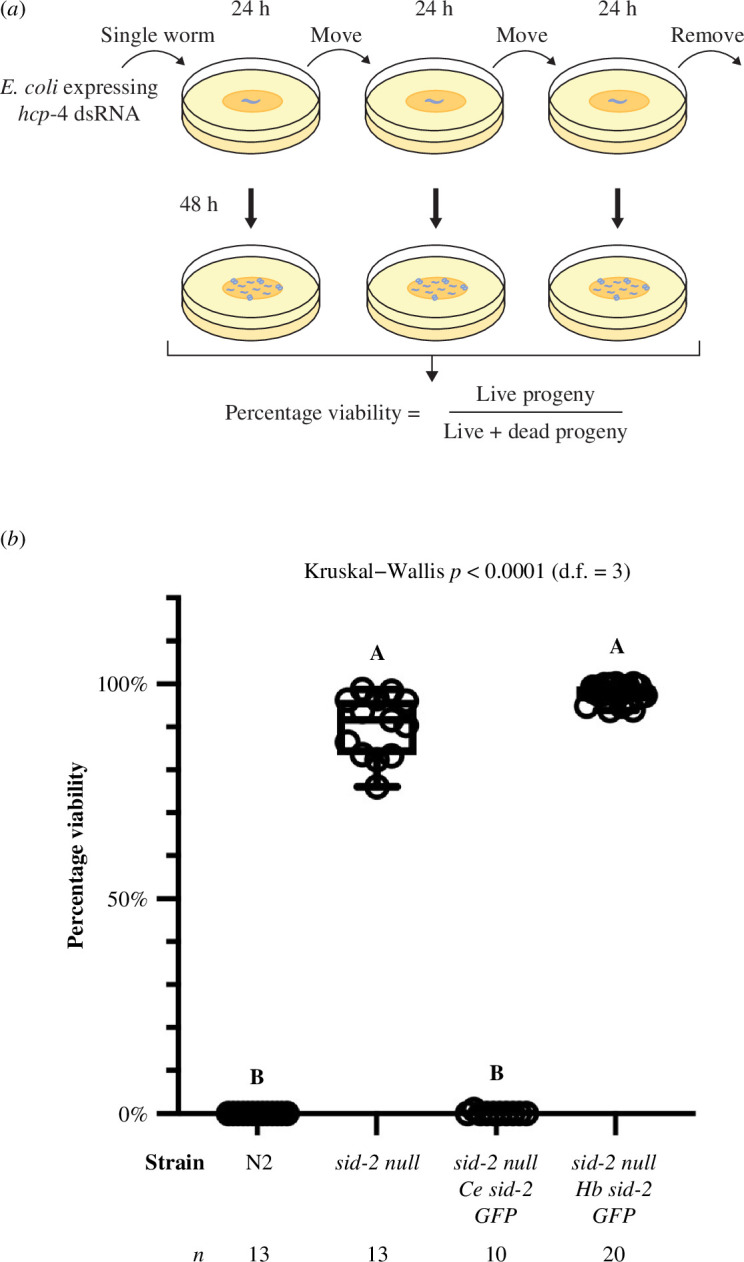
Assay for rescue of *C. elegans* SID-2 function in environmental RNAi. (*a*) Schematic of the environmental RNAi assay. (*b*) Percentage viability of offspring from SID-2 transgenic worms fed on bacteria expressing embryonic lethal dsRNA against *hcp-4*. Letters indicate significant differences between strains as determined by Kruskal–Wallis (*p *< 0.0001, d.f. = 3) and Dunn’s post hoc tests. The sample size (*n*) assayed for each strain is displayed below the x-axis.

To test the capacity of *H. bakeri* SID-2 to rescue *C. elegans* SID-2 function in environmental RNAi, we performed an embryonic lethal RNA interference assay. We exposed wild-type N2, SID-2 null, HbSID-2::GFP and CeSID-2::GFP strains to *Escherichia coli* OP-50 expressing dsRNA against *C. elegans* holocentric protein-4 (*hcp-4*), which performs an essential function in chromosome segregation during mitosis [48] and causes embryonic lethality when knocked-down by RNAi [36] (figure 4*a*). Offspring viability was significantly different between strains (Kruskal–Wallis = 46.95, *p *< 0.0001, d.f. = 3), with Dunn’s post hoc tests indicating significant differences between strains with *C. elegans* SID-2 (wild-type N2 and CeSID-2::GFP) and without *C. elegans* SID-2 (SID-2 null and HbSID-2::GFP) (figure 4*b*). As expected, the proportion of viable offspring was 0% in wild-type N2 and CeSID-2::GFP transgenic worms, consistent with functional uptake of lethal dsRNA from the intestinal lumen by SID-2, and subsequent entry into RNAi pathways in the worm. In the SID-2 null mutant, the median proportion of viable offspring was 91.6%, indicating significantly reduced efficacy of RNAi similar to what was seen with HbSID-2::GFP transgenic worms (98.1%). These results demonstrate that *H. bakeri* SID-2 does not internalize dsRNA from the intestinal lumen, or that internalized dsRNA does not enter functional RNAi pathways (figure 4*b*).

## 4. Discussion

Despite a lack of environmental RNAi phenotypes and failure to find orthologues of *C. elegans sid-2* in previous searches [11,12], our results show that *sid-2* is present in syntenic regions of clade V parasite genomes. The highest conservation is seen in the transmembrane and cytoplasmic domains, and our analysis highlights the divergence of the extracellular domain of SID-2, which shows very little conservation across the clade V nematode orthologues examined. A previous mutagenesis study pinpointed a requirement of the SID-2 extracellular domain for internalization of dsRNA from the environment: the extracellular domain of *C. elegans* SID-2 was able to rescue an environmental RNAi phenotype in a *sid-2 null* mutant, whereas the divergent extracellular domain from *C. briggsae* SID-2, a species that does not exhibit an environmental RNAi phenotype, does not rescue the phenotype [5]. The high divergence of the extracellular domain may explain the multiple gains and losses of environmental RNAi phenotypes in the *Caenorhabditis* genus, despite all of these species encoding *sid-2* orthologues [6]. The lack of conservation of the extracellular domain is consistent with our finding here that *H. bakeri* SID-2 does not rescue RNAi in *C. elegans* and is unlikely to operate in dsRNA import in the parasite (where environmental RNAi by feeding or soaking in dsRNA has not been shown to work [10]).

The mechanism of dsRNA import by SID-2, and the role of the extracellular domain in this process, is not yet fully understood, but it is proposed to function by pH-dependent receptor-mediated endocytosis [5]. In this regard, it is expected that the extracellular domain of SID-2 acts as a receptor, whereas the cytoplasmic domain interacts with downstream partners in the endocytosis pathway. Here we show that the cytoplasmic domain is relatively conserved across the nematodes which could indicate a common function in endocytosis, but we do not know what molecules/substrates might be internalized by SID-2. In addition to potential variation in the substrate interactions of SID-2 orthologues, a key implication from this work is that the function of SID-2 could extend well beyond the apical membrane of the intestine. Our proteomic and western blot analysis shows that SID-2 is highly abundant in extracellular vesicles released from the parasite, which we have previously shown interact with mammalian host cells [15–17]. Recent reports have also demonstrated that some EVs released from *C. elegans* contain SID-2 [13]. An open question is whether the divergent extracellular domain of SID-2 could play a dynamic role in the interaction and uptake of EVs by cells and whether it retains the ability to bind any form of RNA. Together these studies expand the contexts in which we should consider SID-2 and its potential interaction partners, and how they may function in the interactions between nematodes and their environments.

## 5. Conclusion

We identified an orthologue of *C. elegans sid-2* that is highly enriched in *H. bakeri* EVs. Using genome synteny and predicted protein structure-based comparisons, we identified *sid-2* orthologues in other clade V parasitic nematodes. An evolutionary conservation analysis of the protein sequences (using ConSurf) showed that the extracellular domain of SID-2 orthologues in clade V nematodes, which is essential for the environmental RNAi phenotype in *C. elegans*, is more divergent than the transmembrane and cytoplasmic domains. Consistent with previous findings that *H. bakeri* does not exhibit an environmental RNAi phenotype *in vitro*, transgenic expression of *H. bakeri* SID-2 in a *C. elegans* null mutant did not compensate for *C. elegans* SID-2 function in environmental RNAi. We speculate that an ancestral function of nematode SID-2 proteins could relate to EV-mediated communication in free-living and parasitic nematodes.

## Data Availability

The datasets supporting this article have been uploaded as part of the supplementary material [49].

## References

[1] Whangbo JS, Hunter CP. 2008 Environmental RNA interference. Trends Genet. **24**, 297–305. (10.1016/j.tig.2008.03.007) 18450316

[2] Tijsterman M, May RC, Simmer F, Okihara KL, Plasterk RHA. 2004 Genes required for systemic RNA interference in Caenorhabditis elegans. Curr. Biol. **14**, 111–116. (10.1016/j.cub.2003.12.029) 14738731

[3] Winston WM, Molodowitch C, Hunter CP. 2002 Systemic RNAi in C. elegans requires the putative transmembrane protein SID-1. Science **295**, 2456–2459. (10.1126/science.1068836) 11834782

[4] Winston WM, Sutherlin M, Wright AJ, Feinberg EH, Hunter CP. 2007 Caenorhabditis elegans SID-2 is required for environmental RNA interference. Proc. Natl Acad. Sci. USA **104**, 10565–10570. (10.1073/pnas.0611282104) 17563372 PMC1965553

[5] McEwan DL, Weisman AS, Hunter CP. 2012 Uptake of extracellular double-stranded RNA by SID-2. Mol. Cell **47**, 746–754. (10.1016/j.molcel.2012.07.014) 22902558 PMC3488460

[6] Nuez I, Félix MA. 2012 Evolution of susceptibility to ingested double-stranded RNAs in Caenorhabditis nematodes. PLoS One **7**, e29811. (10.1371/journal.pone.0029811) 22253787 PMC3256175

[7] Blaxter ML *et al*. 1998 A molecular evolutionary framework for the phylum Nematoda. Nature **392**, 71–75. (10.1038/32160) 9510248

[8] Smythe AB, Holovachov O, Kocot KM. 2019 Improved phylogenomic sampling of free-living nematodes enhances resolution of higher-level nematode phylogeny. BMC Evol. Biol. **19**, 121. (10.1186/s12862-019-1444-x) 31195978 PMC6567515

[9] Maizels RM *et al*. 2012 Immune modulation and modulators in Heligmosomoides polygyrus infection. Exp. Parasitol. **132**, 76–89. (10.1016/j.exppara.2011.08.011) 21875581 PMC6485391

[10] Lendner M, Doligalska M, Lucius R, Hartmann S. 2008 Attempts to establish RNA interference in the parasitic nematode Heligmosomoides polygyrus. Mol. Biochem. Parasitol. **161**, 21–31. (10.1016/j.molbiopara.2008.06.003) 18606194

[11] Viney ME, Thompson FJ. 2008 Two hypotheses to explain why RNA interference does not work in animal parasitic nematodes. Int. J. Parasitol. **38**, 43–47. (10.1016/j.ijpara.2007.08.006) 18028931

[12] Dalzell JJ *et al*. 2011 RNAi effector diversity in nematodes. PLoS Negl. Trop. Dis. **5**, e1176. (10.1371/journal.pntd.0001176) 21666793 PMC3110158

[13] Nikonorova IA *et al*. 2022 Isolation, profiling, and tracking of extracellular vesicle cargo in Caenorhabditis elegans. Curr. Biol. **32**, 1924–1936. (10.1016/j.cub.2022.03.005) 35334227 PMC9491618

[14] Wang J, Silva M, Haas LA, Morsci NS, Nguyen KCQ, Hall DH, Barr MM. 2014 C. elegans ciliated sensory neurons release extracellular vesicles that function in animal communication. Curr. Biol. **24**, 519–525. (10.1016/j.cub.2014.01.002) 24530063 PMC4659354

[15] Buck AH *et al*. 2014 Exosomes secreted by nematode parasites transfer small RNAs to mammalian cells and modulate innate immunity. Nat. Commun. **5**, 5488. (10.1038/ncomms6488) 25421927 PMC4263141

[16] Coakley G *et al*. 2017 Extracellular vesicles from a helminth parasite suppress macrophage activation and constitute an effective vaccine for protective immunity. Cell Rep. **19**, 1545–1557. (10.1016/j.celrep.2017.05.001) 28538175 PMC5457486

[17] Chow FWN *et al*. 2019 Secretion of an argonaute protein by a parasitic nematode and the evolution of its siRNA guides. Nucleic Acids Res. **47**, 3594–3606. (10.1093/nar/gkz142) 30820541 PMC6468290

[18] White R *et al*. 2023 Special considerations for studies of extracellular vesicles from parasitic helminths: a community-led roadmap to increase rigour and reproducibility. J. Extracell. Vesicles **12**, e12298. (10.1002/jev2.12298) 36604533 PMC9816087

[19] Hallgren J, Tsirigos KD, Pedersen MD, Almagro Armenteros JJ, Marcatili P, Nielsen H, Krogh A, Winther O. 2022 DeepTMHMM predicts alpha and beta transmembrane proteins using deep neural networks. Bioinformatics 2022. (10.1101/2022.04.08.487609)35134862

[20] Abramson J *et al*. 2024 Accurate structure prediction of biomolecular interactions with AlphaFold 3. Nature **630**, 493–500. (10.1038/s41586-024-07487-w) 38718835 PMC11168924

[21] Gupta R, Brunak S. 2002 Prediction of glycosylation across the human proteome and the correlation to protein function. In Pacific symposium on biocomputing 2002 (eds RB Altman, AK Dunker, L Hunter, K Lauderdale, TE Klein), pp. 310–322. Singapore: World Scientific. (10.1142/9789812799623_0029) 11928486

[22] Howe KL, Bolt BJ, Shafie M, Kersey P, Berriman M. 2017 WormBase Parasite—a comprehensive resource for helminth genomics. Mol. Biochem. Parasitol. **215**, 2–10. (10.1016/j.molbiopara.2016.11.005) 27899279 PMC5486357

[23] Li H. 2023 Protein-to-genome alignment with miniprot. Bioinformatics **39**. (10.1093/bioinformatics/btad014) PMC9869432 36648328

[24] Davis P *et al*. 2022 WormBase in 2022-data, processes, and tools for analyzing Caenorhabditis elegans. Genetics **220**, iyac003. (10.1093/genetics/iyac003) 35134929 PMC8982018

[25] Yariv B, Yariv E, Kessel A, Masrati G, Chorin AB, Martz E, Mayrose I, Pupko T, Ben-Tal N. 2023 Using evolutionary data to make sense of macromolecules with a 'face-lifted' ConSurf. Protein Sci. **32**, e4582. (10.1002/pro.4582) 36718848 PMC9942591

[26] Madeira F, Pearce M, Tivey ARN, Basutkar P, Lee J, Edbali O, Madhusoodanan N, Kolesnikov A, Lopez R. 2022 Search and sequence analysis tools services from EMBL-EBI in 2022. Nucleic Acids Res. **50**, W276–W279. (10.1093/nar/gkac240) 35412617 PMC9252731

[27] RStudio Team. 2021 RStudio: integrated development environment for R. See https://github.com/rstudio/rstudio.

[28] R Core Team. 2019 R: a language and environment for statistical computing. Vienna, Austria: R Foundation for Statistical Computing.

[29] Wickham H. 2016 Ggplot2: elegant graphics for data analysis. Berlin, Germany: Springer. (10.1007/978-3-319-24277-4_9)

[30] Pedersen TL. 2024 Ggforce: accelerating ‘ggplot2. See https://cran.r-project.org/web/packages/ggforce/ggforce.pdf.

[31] Wickham H, Vaughan D, Girlich M. 2024 Tidyr: tidy messy data. See https://cran.r-project.org/web/packages/tidyr/tidyr.pdf.

[32] Yu G, Smith DK, Zhu H, Guan Y, Lam TY. 2017 Ggtree: an r package for visualization and annotation of phylogenetic trees with their covariates and other associated data. Methods Ecol. Evol. **8**, 28–36. (10.1111/2041-210X.12628)

[33] Wang LG *et al*. 2020 Treeio: an r package for phylogenetic tree input and output with richly annotated and associated data. Mol. Biol. Evol. **37**, 599–603. (10.1093/molbev/msz240) 31633786 PMC6993851

[34] Frøkjaer-Jensen C, Davis MW, Hopkins CE, Newman BJ, Thummel JM, Olesen SP, Grunnet M, Jorgensen EM. 2008 Single-copy insertion of transgenes in Caenorhabditis elegans. Nat. Genet. **40**, 1375–1383. (10.1038/ng.248) 18953339 PMC2749959

[35] Gibson DG, Young L, Chuang RY, Venter JC, Hutchison CA 3rd, Smith HO. 2009 Enzymatic assembly of DNA molecules up to several hundred kilobases. Nat. Methods **6**, 343–345. (10.1038/nmeth.1318) 19363495

[36] Taylor SJP, Bel Borja L, Soubigou F, Houston J, Cheerambathur DK, Pelisch F. 2023 BUB-1 and CENP-C recruit PLK-1 to control chromosome alignment and segregation during meiosis I in C. elegans oocytes. eLife **12**. (10.7554/eLife.84057) PMC10156168 37067150

[37] Timmons L, Court DL, Fire A. 2001 Ingestion of bacterially expressed dsRNAs can produce specific and potent genetic interference in Caenorhabditis elegans. Gene **263**, 103–112. (10.1016/s0378-1119(00)00579-5) 11223248

[38] Altschul SF, Gish W, Miller W, Myers EW, Lipman DJ. 1990 Basic local alignment search tool. J. Mol. Biol. **215**, 403–410. (10.1016/S0022-2836(05)80360-2) 2231712

[39] Carlton PM, Davis RE, Ahmed S. 2022 Nematode chromosomes. Genetics **221**, iyac014. (10.1093/genetics/iyac014) 35323874 PMC9071541

[40] Stevens L *et al*. 2023 Ancient diversity in host–parasite interaction genes in a model parasitic nematode. Nat. Commun. **14**, 7776. (10.1038/s41467-023-43556-w) 38012132 PMC10682056

[41] van Kempen M, Kim SS, Tumescheit C, Mirdita M, Lee J, Gilchrist CLM, Söding J, Steinegger M. 2024 Fast and accurate protein structure search with Foldseek. Nat. Biotechnol. **42**, 243–246. (10.1038/s41587-023-01773-0) 37156916 PMC10869269

[42] Krissinel E, Henrick K. 2004 Secondary-structure matching (SSM), a new tool for fast protein structure alignment in three dimensions. Acta Cryst. D Biol. Cryst. **60**, 2256–2268. (10.1107/S0907444904026460) 15572779

[43] Agirre J *et al*. 2023 The CCP4 suite: integrative software for macromolecular crystallography. Acta Crystallogr. D. Struct. Biol. **79**, 449–461. (10.1107/S2059798323003595) 37259835 PMC10233625

[44] Rath A, Glibowicka M, Nadeau VG, Chen G, Deber CM. 2009 Detergent binding explains anomalous SDS-PAGE migration of membrane proteins. Proc. Natl Acad. Sci. USA **106**, 1760–1765. (10.1073/pnas.0813167106) 19181854 PMC2644111

[45] Magnelli PE, Bielik AM, Guthrie EP. 2011 Identification and characterization of protein glycosylation using specific endo- and exoglycosidases. J. Vis. Exp. **58**, e3749. (10.3791/3749) PMC3369641 22230788

[46] Ashkenazy H, Erez E, Martz E, Pupko T, Ben-Tal N. 2010 ConSurf 2010: calculating evolutionary conservation in sequence and structure of proteins and nucleic acids. Nucleic Acids Res. **38**, W529–33. (10.1093/nar/gkq399) 20478830 PMC2896094

[47] Ashkenazy H, Abadi S, Martz E, Chay O, Mayrose I, Pupko T, Ben-Tal N. 2016 ConSurf 2016: an improved methodology to estimate and visualize evolutionary conservation in macromolecules. Nucleic Acids Res. **44**, W344–50. (10.1093/nar/gkw408) 27166375 PMC4987940

[48] Moore LL, Roth MB. 2001 HCP-4, a CENP-C-like protein in Caenorhabditis elegans, is required for resolution of sister centromeres. J. Cell Biol. **153**, 1199–1208. (10.1083/jcb.153.6.1199) 11402064 PMC2192019

[49] Blow F, Jeffrey K, Chow FWN, Nikonorova IA, Barr MM, Cook AG. 2024 Data from: SID-2 is a conserved extracellular vesicle protein that is not associated with environmental RNAi in parasitic nematodes. Figshare. (10.6084/m9.figshare.c.7484140) PMC11538922 39501794

